# Arrhythmogenic mitral valve prolapse and mitral annular disjunction: a literature review and case-based perspective

**DOI:** 10.3389/fcvm.2025.1698603

**Published:** 2025-11-19

**Authors:** Austė Markevičiūtė, Sandra Kmitaitė, Audronė Vaitiekienė, Diana Rinkūnienė

**Affiliations:** 1 Faculty of Medicine, Medical Academy, Lithuanian University of Health Sciences, Kaunas, Lithuania; 2 Department of Cardiology, Lithuanian University of Health Sciences Kaunas Clinics, Kaunas, Lithuania

**Keywords:** mitral valve prolapse, mitral annular disjunction, ventricular arrhythmias, echocardiography, cardiac magnetic resonance

## Abstract

Mitral valve prolapse (MVP) is among the most frequently diagnosed valvular heart diseases, with the majority of patients being asymptomatic. However, MVP may also present with ventricular arrhythmias (VA) or sudden cardiac death (SCD). The arrhythmic form of MVP and the complicated course of this condition are closely associated with mitral annular disjunction (MAD). MAD alters the anatomical integrity of the mitral annulus and is thought to contribute to leaflet degeneration and myocardial fibrosis, creating a substrate for malignant arrhythmias. The most common complications associated with MVP and MAD include progressive mitral valve regurgitation, left ventricular hypertrophy and an increased risk of SCD. Cardiac magnetic resonance (CMR) imaging is the most sensitive modality for detecting MAD and myocardial fibrosis and is thus essential for early diagnosis and risk stratification. This review aims to enhance recognition of MAD, summarize current diagnostic and risk assessment strategies, and provide clinical context through two cases of patients with arrhythmic MVP and MAD.

## Introduction

Mitral valve prolapse (MVP) is among the most frequently diagnosed heart valve diseases, with a prevalence of 2%–4% among the general population (1). This condition is described as the displacement of one or both mitral valve (MV) leaflets into the left atrium (LA) during systole, which may also cause mitral valve regurgitation (MVR) (2). While MVP is commonly considered to be a benign condition and many patients are asymptomatic, a small subset of patients may develop complications such as ventricular arrhythmias (VA) or even sudden cardiac death (SCD). Arrhythmic events in presence of MVP without a significant MVR may arise because of morphological valve changes or myocardial fibrosis, as the excessive leaflet motion during systole induces abnormal stretch on the papillary muscles and adjacent inferolateral ventricular wall, causing microinjury to the myocardium and subsequent fibrotic remodeling, forming a substrate for ventricular ectopy and re-entry circuits (3). These morphological alterations form the basis of the emerging term “malignant” or “arrhythmic” MVP (AMVP), which is used to describe this phenotype's particular predisposition to VAs. In addition, one of the main factors, associated with a complicated MVP course, is thought to be mitral annular disjunction (MAD) (4). This structural abnormality is found in nearly 30% of MVP patients and is characterized by the separation of mitral annulus and its' posterior leaflet from ventricular myocardium into the left atrium during systole (5). This, consequently, puts a strain on the chordae tendineae of the MV, causing their stretching and degeneration.

Although MAD can be observed during routine two-dimensional transthoracic echocardiography (TTE), cardiac magnetic resonance (CMR) imaging is considered the gold standard for diagnosing this structural pathology. It enables the detection of the three key components that are most strongly associated with AMVP: MAD, myocardial fibrosis detected as late gadolinium enhancement (LGE) and impaired global longitudinal strain. Identification of any single abnormality among these findings increases the likelihood of the development of VAs and even SDC. Early diagnosis, as well as further risk stratification, is crucial to prevent the development and progression of MVR and life-threatening arhythmic events.

The aim of this review is to enhance recognition of MAD, highlighting its' clinical significance and providing an overview of its differential diagnosis, diagnostics, treatment options, and risk stratification in the presence of arrhythmic MVP. Additionally, we present two illustrative case reports of patients diagnosed with both MAD and MVP.

## Clinical cases

### Clinical case 1

An 18-year-old female patient with a history of MVP and recurrent episodes of VA presented to a tertiary care center for a cardiology consultation. The patient complained of recurrent episodes of tachycardia and palpitations, which worsened during physical exercise. The patient presented to the clinic in 2017, when an ablation of the accessory pathway (Wolff-Parkinson-White) was performed and treatment with Metoprolol was initiated, after which the symptoms subsided. However, in recent years, the patient started complaining of an increased frequency of palpitations. A 24 h Holter monitor (HM) was performed and revealed that 3% of all recorded heartbeats were premature ventricular contractions (PVCs), with several episodes of ventricular bigeminy. The morphology of PVCs was predominantly monomorphic, showing left bundle branch block (LBBB) with an inferior axis, suggesting possible origin from the papillary muscle region or the basal inferolateral LV wall—a pattern frequently seen in arrhythmic MVP. However, resting 12-lead ECG did not demonstrate biphasic or negative T-waves in the inferior-lateral leads.

**Figure 1:**
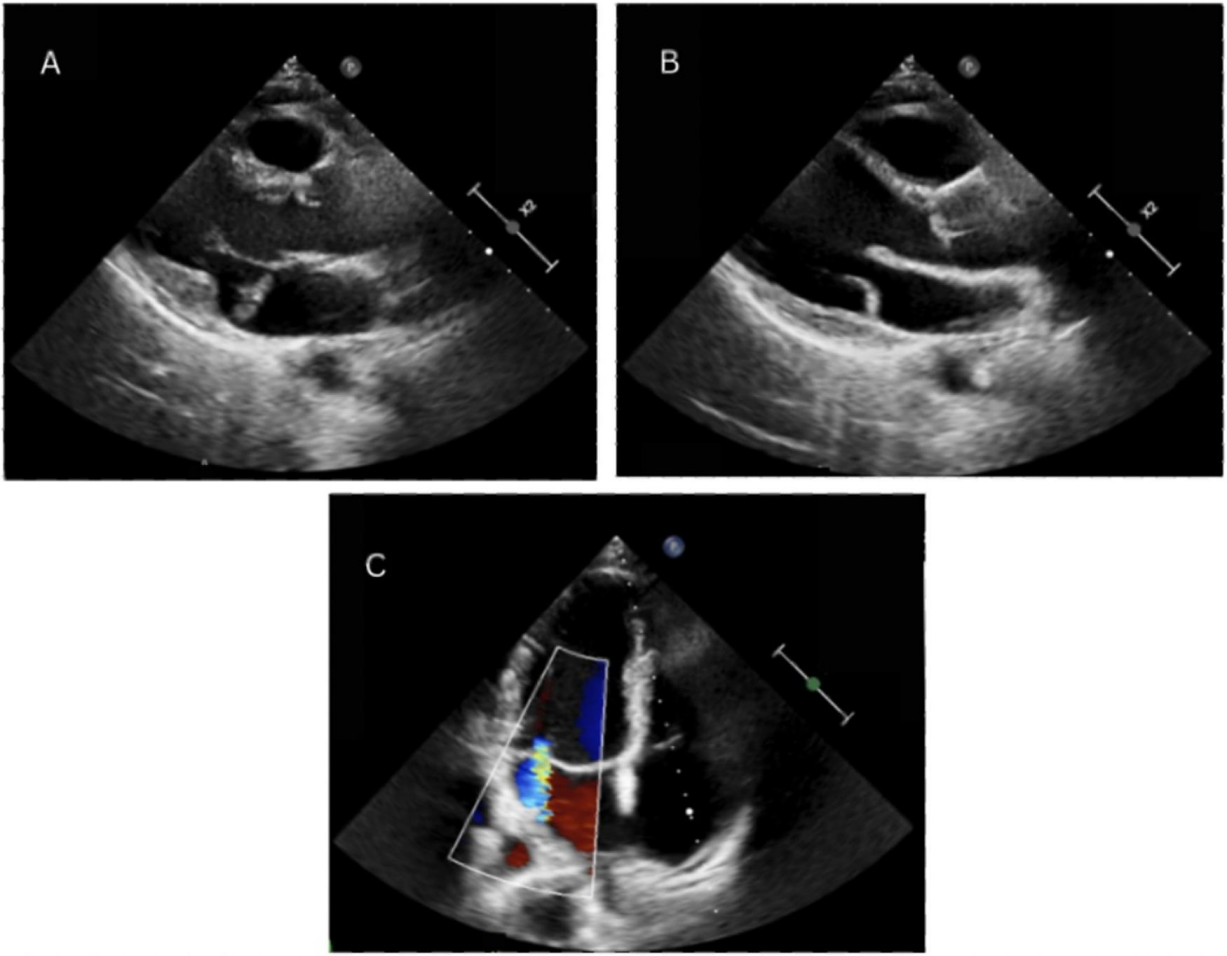
Echocardiography demonstrating mitral annular disjunction. Parasternal long-axis view obtained at end-systole and diastole, showing the separation between the posterior mitral valve leaflet insertion point and the left ventricular myocardium **(A,B)**. Color Doppler imaging visualizing mitral regurgitation, evidenced by a central regurgitant jet **(C)**.

TTE was also performed to reveal normal diameters and functions of all heart chambers, bileaflet MVP and moderate MVR, which can be seen in Figure 1. To rule out underlying structural heart disease, CMR was performed, which confirmed MVP with moderate MVR, also revealing the presence of MAD. No LGE was detected, indicating the absence of myocardial fibrosis. Subsequently, the patient was hospitalized for three days for a 72 h HM and treatment planning. During the hospitalization no episodes of ventricular tachycardia (VT) were recorded, and the daily burden of PVCs ranged between 600 and 800. The patient's medical data were reevaluated by a team of electrophysiologists who concluded that, despite the absence of myocardial fibrosis and no currently documented episodes of VT, long-term rhythm monitoring was necessary. This decision was based on the 2022 ESC guidelines, which support ILR implantation (Class IIa) in patients with a history of unexplained syncope or prior VAs, even in the absence of conventional high-risk markers (6). In this case, ILR implantation was justified by the patient's history of previously recorded VT episodes and syncope, her engagement in regular intense physical activity, and the presence of arrhythmogenic mitral valve features (including MAD and PVC morphology suggestive of papillary muscle origin).

### Clinical case 2

A 51-year-old female with a medical history of spinal cord trauma, MVP and dyslipidemia was referred to a tertiary care center for further assessment of the etiology and mechanism of MVR, as well as evaluation for suspected cardiomyopathy. She had a family history of early–onset cardiovascular disorders—both sisters experienced myocardial infarction at ages 38 and 36, her mother suffered a stroke and her father died from SCD at the age of 40. The patient was also a smoker, with a reported smoking history of 15–20 years, smoking 15 cigarettes per day.

**Figure 2:**
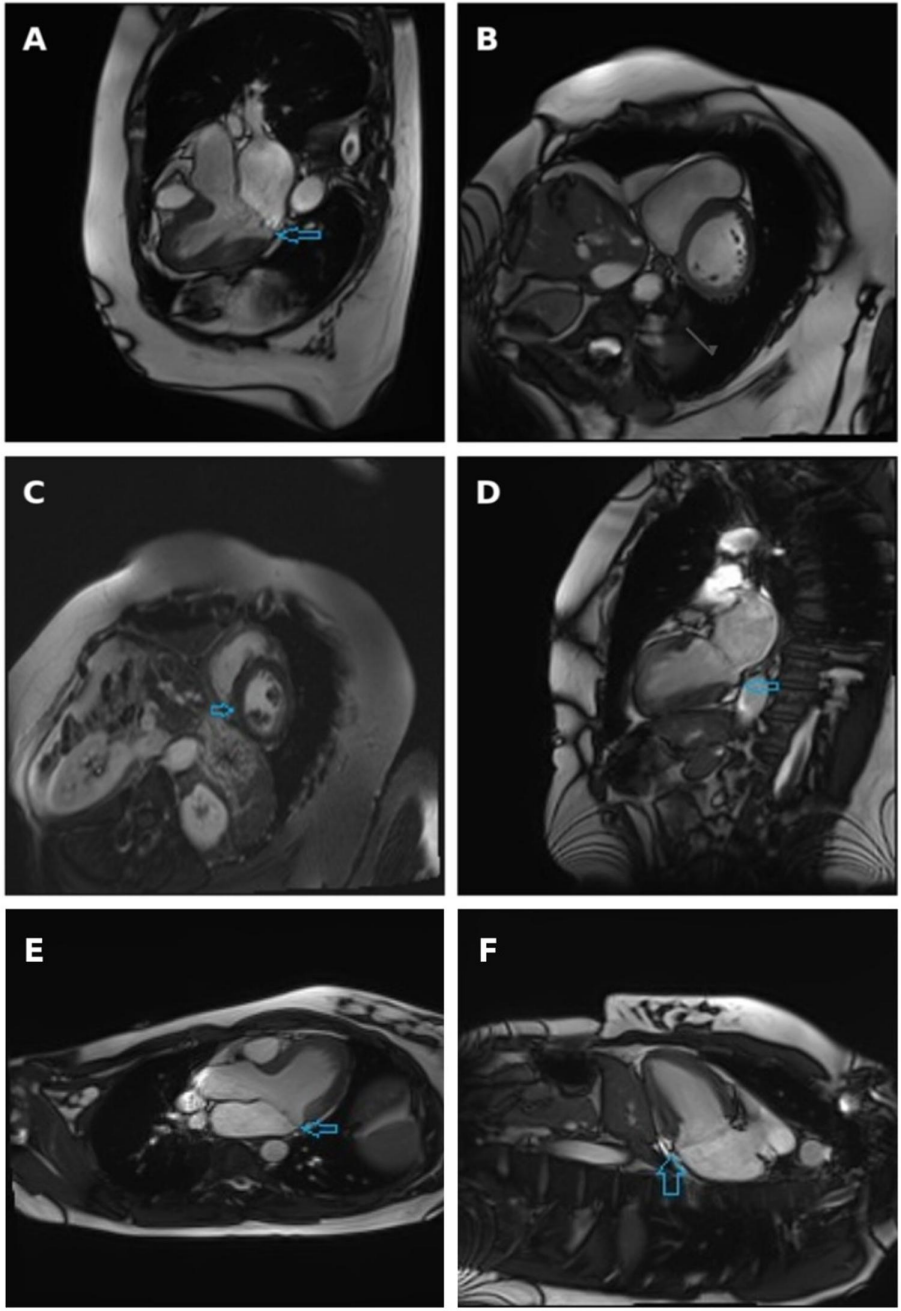
Cine three-chamber long-axis view **(A)**, short-axis view at basal segments level **(B)** and two-chamber view **(D)** obtained at systole, with arrows indicating the separation between the posterior mitral valve leaflet insertion and the left ventricular myocardium. Late gadolinium enhancement short-axis view: arrow indicates non-specific fibrosis at the inferior right ventricular insertion point **(C)** Cardiac magnetic resonance imaging demonstrating mitral annular disjunction. Three-chamber long-axis view and **(E)** two-chamber view **(F)** obtained at end-systole, with arrows indicating the separation between the posterior mitral valve leaflet insertion point and the left ventricular myocardium.

A comprehensive TTE revealed a thickened intraventricular septum, anterior MV leaflet prolapse, myxomatous degeneration of the MV leaflets, moderate to severe MVR with two eccentric jets, as well as LA and mitral annulus dilatation. Coronary artery angiography revealed mild non-obstructive coronary artery disease. CMR revealed inferior right ventricular insertion point fibrosis, moderate to severe MVR and MAD, all of which can be seen in Figure 2. Although LGE was detected, its distribution was located at the right ventricular insertion point, a site where enhancement is often considered non-specific and not necessarily indicative of a malignant arrhythmogenic substrate. Such findings, if isolated, should therefore be interpreted with caution, particularly in the absence of additional high-risk imaging features. However, additional Holter monitoring in this patient revealed polymorphic PVCs, several episodes of bigeminy, trigeminy and couplets. The PVCs exhibited both LBBB and right bundle branch block (RBBB) morphologies, suggesting multiple ectopic foci, most likely originating from the papillary muscles and/or adjacent inferior-basal LV myocardium. Moreover, a 12-lead ECG showed deep T-wave inversions in the inferior and lateral leads, which further supported the arrhythmogenic substrate and higher risk profile for this patient. The patient was discharged with a recommended adjustment of beta-blocker therapy and a plan for continued outpatient monitoring. A follow-up visit to a cardiologist was scheduled after 6 months, with 48 h HM and TTE assessment to be performed.

After a subsequent visit to a cardiologist, a 48 h HM revealed no episodes of VT. However, the daily burden of polymorphic PVCs was approximately 11,000. The patient's medical data were reevaluated by a team of electrophysiologists, who concluded that long-term rhythm monitoring was indicated despite the absence of sustained VT and conventional high-risk imaging findings. This decision was based on the presence of multiple phenotypic risk features, including a very high daily PVC burden (>11,000), polymorphic morphology indicating multiple ectopic foci, deep T-wave inversions in the inferior–lateral leads and a strong family history of premature cardiovascular disease and SCD, which, combined, place this patient in the intermediate-risk category.

## Discussion

### Clinical presentation

In a systematic review, Putnam and colleagues stated that up to one-third of patients diagnosed with MVP and/or MAD remain asymptomatic (7). However, the majority of patients experience symptoms, among which the most common ones are palpitations and unexplained syncope or pre-syncope episodes. These symptoms are often accompanied by other non-specific manifestations, such as dyspnea, fatigue, reduced exercise capacity, and chest discomfort. Although the distribution of MVP by gender is relatively equal, a retrospective study conducted by Gray et al. examined 111 patients who underwent surgical correction of MVP and found that the prevalence of MAD was greater in the female population compared to the male population (8).

Thus, because of its diverse clinical manifestations, ranging from asymptomatic cases to subtle non-specific symptoms, the diagnosis of MAD is often underrecognized.

### Diagnostic

A mid-systolic click and/or a late systolic murmur can be heard during cardiac auscultation, suggestive of MVP. Such findings indicate the need for a more detailed diagnostic evaluation.

While electrocardiography (ECG) alone cannot confirm or deny both MVP or MAD, certain ECG features, such as inverted or biphasic T waves in the posterior-lateral leads, may be observed in patients with MVP and MAD (9). MAD is also often associated with inflammation of myocardium and left ventricular (LV) fibrosis, which may lead to the development of VAs, including PVCs, monomorphic and polymorphic VT episodes or even ventricular fibrillation (VF).

However, the gold standard diagnostic imaging tool for identifying and confirming the diagnosis of MVP is two-dimensional TTE. The bowing of MV leaflets is best visualized in the parasternal long-axis (PLAX) and apical three-chamber views during systole. In the PLAX view, the posterior and/or anterior leaflet displacement into the LA by at least 2 mm during systole must be observed to meet the diagnostic criteria for MVP (10). Other findings, common in MVP, include the thickening of leaflets, best observed in PLAX view during mid-diastole and an increased size of the LA, usually resulting from severe MVR (11). In a systematic review, conducted in 2022 by Pype and colleagues, it was noted that remodeling and dilatation of LV was more frequently seen in MVP patients when compared to the healthy population. In the studied population with MVP, LV dimensions were also not dependent on the degree of MVR (12). A continuous-wave Doppler can also be used to evaluate the regurgitation jet direction during late systole, which, in case of MVP, will be directed opposite to the bowing leaflet. However, if both MV leaflets are prolapsing, then a central regurgitative jet will be observed.

MAD is best visualized in the TTE PLAX view and is often accompanied by myxomatous damage of MV leaflets. However, the most reliable and accurate tool used for the evaluation of fibrosis of myocardium and detection of MAD is CMR, as demonstrated in Figure 2. In a systematic literature review conducted by Drescher and colleagues, the diagnostic sensitivity of TTE and CMR was compared in the detection of MAD. MAD was detected in 17.3% of individuals using TTE, whereas the prevalence of MAD increased to 42.0% when the same individuals were assessed using CMR (13). To fully assess the extent of MAD, six LV long-axis cine sequences with 30° interslice rotation must be acquired around the mitral annulus, evaluating its' circumferential extent and maximal width of the disjunction (14). Another advantage of CMR compared to TTE is the ability to evaluate focal fibrosis of myocardium using LGE as well as diffuse LV fibrosis, which is associated with an increased risk of VAs, determined by T1 mapping (15). In presence of MAD, fibrosis is usually located close to the mitral annulus in the basal LV wall, including papillary muscles and inferior LV wall. Another common finding in patients with MAD is tricuspid annular disjunction (TAD), which, according to a study by Aabel et al., has been reported to occur in approximately 50% of examined MAD cases. The coexistence of TAD and MAD suggests a broader involvement of the atrioventricular junction rather than an isolated MV pathology and has been associated with a greater increase in the arrhythmic risk. Although standard CMR protocols can visualize the tricuspid annulus, TAD still often remains underdiagnosed (16). However, in the two cases presented in this study, no evidence of concomitant TAD was found on CMR.

While imaging findings play a crucial role in identifying structural abnormalities, the most important tool for risk stratification and selecting the appropriate treatment plan is HM as well as ILR, which allows for long-term rhythm monitoring. ILR implantation is usually chosen in high or moderate-risk patient groups, who experience syncope episodes, have had episodes of VT in the past, had LGE seen on CMR or presented with inconclusive HM data (14).

### Risk stratification

Based on the Holter monitoring results, patients are classified into high, intermediate and low risk groups, and the individual treatment plan is chosen accordingly. According to the data published by the EHRA committee in 2022, the high-risk group includes: sustained VT lasting more than 30 s or requiring immediate termination by external means, spontaneous polymorphic non-sustained ventricular tachycardia (NSVT) lasting 30 s or less and monomorphic NSVT with a ventricular rate exceeding 180 bpm. The intermediate risk group includes frequent polymorphic PVCs, monomorphic NSVT with a ventricular rate below 180 bpm and registered complex VAs, such as ventricular bigeminy and couplets. Patients with isolated frequent PVCs are considered to be in a low-risk group (14).

In addition to rhythm-based stratification, recent imaging and ECG data have provided new insights into the understanding of arrhythmic risk in MVP. In a recent meta-analysis by Pistelli et al., which included 1,715 patients with MVP, CMR finding of LGE was among the strongest predictors of arrhythmic events, with an odds ratio (OR) of approximately 16.7. The study further demonstrated that patients with arrhythmic MVP had significantly MAD length (+1.24 mm), thicker anterior and posterior mitral leaflets (+0.49 mm and +2.96 mm, respectively), and increased anterior mitral leaflet length (+2.63 mm) (17). These results suggest that integrating detailed morphological assessment from multimodality imaging may improve the accuracy of risk stratification, regardless of arrhythmic symptoms or the degree of MVR.

Moreover, data from the same systematic review and meta-analysis emphasize that even in minimally symptomatic or asymptomatic MVP patients, structural and ECG abnormalities carry measurable prognostic value. LGE, MAD length, bileaflet prolapse morphology, and inferolateral T-wave inversion were identified as key markers that differentiate arrhythmic from non-arrhythmic MVP phenotypes (17). This highlights the need for early multimodality imaging assessment and integrated evaluation combining structural, functional, and electrical parameters to optimize patient risk classification.

### Treatment

Pharmacological therapy in cases of asymptomatic MVP is not recommended. When arrhythmic symptoms are present, patients are typically prescribed beta-blockers. It is proven that pharmacological treatment can alleviate symptoms and improve LV function. However, it has little effect on the frequency of VAs. It is important to note that in patients prone to bradyarrhythmia episodes beta-blockers may cause life-threatening bradycardia, and thus their use in such patient groups is contraindicated. In recent years, the potential role of class IC antiarrhythmic drugs, particularly flecainide, has been recognized for the treatment of VAs of idiopathic or mitral origin. Although beta-blockers still remain first-line pharmacological treatment, they often fail to sufficiently suppress VA events. In a recent study conducted by Aabel et al, flecainide, either as monotherapy or combined with beta-blockers, was shown to significantly reduce PVC burden, NSVT episodes, and symptomatic arrhythmias in patients with mitral arrhythmias without significant myocardial fibrosis or underlying structural cardiac diseases. This may be especially relevant in patients similar to those presented in our cases, who exhibit a high PVC burden and arrhythmic symptoms despite preserved LV function and no detectable myocardial fibrosis (18).

One of the interventional treatment options for managing VAs is radiofrequency ablation (RFA). According to the ESC guidelines published in 2022, RFA is recommended if one of the following criteria are met: a patient experiences symptomatic VAs that are resistant to medical therapy, when pharmacological treatment is not tolerated or is contraindicated, also in VA-induced cardiomyopathies. RFA is also indicated for PVC-induced VF or in cases with a history of frequent implantable cardioverter-defibrillator (ICD) activations (14).

Some patients can be eligible for ICD implantation if, despite previous treatments, unexplained syncope episodes or prolonged VT episodes persist. ICD implantation is also indicated if the patient has 2 or more of the following phenotypic risk factors: negative T-waves on ECG in inferior-lateral leads, cardiomyopathy, MAD, LV ejection fraction <50%, and/or dilated LA.

The final, often regarded as controversial, treatment option of arrhythmic MVP is surgical mitral valve repair. According to recent studies, after undergoing mitral valve surgery, the frequency of VAs decreased in 55% of symptomatic patients. However, 19% of the patients who had never experienced VAs before surgery developed them for the first time after the surgical treatment (19). Therefore, given the risk-benefit ratio after complex surgical procedures, the first-line treatment for patients with an arrhythmic form of MVP remains symptom management with pharmacological therapy or RFA.

### Complications and causes of death

One of the main arrhythmogenic MVP and MAD complications is progressive MVR. Some studies suggest that there is no direct correlation between the degree of MVR and reported frequency of VAs or SCD (20). On the contrary, the backflow of blood through a regurgitative valve during systole can cause LA hypertrophy, which, over time, can result in an elevated pulmonary pressure and congestive heart failure.

Other complications that can occur in the presence of MVP include atrial fibrillation, LV hypertrophy and diffuse myocardial fibrosis caused by mechanical stretching due to the prolapsing mitral leaflet. The diffuse fibrosis is often associated with an increased risk of VT and SCD (21). Long-term mechanical stress can also cause a less common but dangerous complication—mitral valve chordal rupture. Although rare, it can lead to life-threatening conditions such as acute MVR, pulmonary oedema or acute heart failure (22).

## Conclusions

1. Mitral annular disjunction in presence of arrhythmogenic mitral valve prolapse may increase the burden of ventricular arrhythmias and the risk of sudden cardiac death.
2. Cardiac magnetic resonance imaging is the most informative diagnostic tool recommended when mitral annular disjunction is suspected and remains the gold standard for confirming the diagnosis.
3. In high-risk arrhythmogenic mitral valve prolapse with concomitant mitral annular disjunction, treatment should be tailored individually, carefully evaluating the benefits of medical and interventional therapies, while avoiding overtreatment or unnecessary interventional procedures.
